# Virtual interdisciplinary collaboration during the COVID-19 pandemic: pain and joy in an international joint university

**DOI:** 10.3389/fpsyg.2023.1184640

**Published:** 2023-07-07

**Authors:** Jinjin Lu

**Affiliations:** Academy of Future Education, Xi'an Jiaotong-Liverpool University, Suzhou, China

**Keywords:** interdisciplinary collaboration, video meeting, remote work, technology, learning management platforms

## Abstract

**Background:**

The COVID-19 pandemic has brought interdisciplinary academics and research students many uncertainties and challenges in adapting to new communication styles. Compared with other academics in the same field, interdisciplinary academics might face more challenges in transitioning from traditional face-to-face communication to virtual communication.

**Objective:**

This study aimed to explore the pain and joy of using Western and Chinese localized communication channels in experienced interdisciplinary academics (*N* = 10) and young research students (*N* = 14) during the pandemic. Among them, 14 are Europeans and 10 are Chinese.

**Method:**

Meeting records and participants' reflective writing were used as qualitative data.

**Results:**

We identified five key themes: two were tied to personal and behavioral issues, two were involved in management issues, and one dealt with topic choice issues.

**Conclusion:**

Considering that virtual interdisciplinary teamwork is likely to continue in the post-pandemic period, it is necessary to implement measures such as technical training and voluntary assistants to help alleviate some of the issues that make virtual meetings difficult for participants. Study limitations and future directions are also discussed.

## 1. Introduction

Interdisciplinary collaboration is not a new topic. In the past few decades, several studies have measured the effectiveness and popularity of virtual and face-to-face communication channels, particularly, during the pandemic period. The COVID-19 pandemic and the resulting lockdown, stay-at-home lifestyle, and quarantine accelerated the trend of virtual interaction and communication. People speedily adopted social media technologies for formal and informal meetings, sharing information, and learning and teaching. Recent studies show that virtual meeting platforms, such as Google Meet, Microsoft Teams, and VooV, saw a significant increase in the number of daily users (Peters, 2020; Thorp-Lancaster, 2020). Scholars (Standaert et al., 2021) believed that virtual meetings will continue and become a widely popular method of communication in interdisciplinary collaboration in the post-pandemic era.

Interdisciplinary collaboration has been examined through the use of different digital technologies (e.g., Fosslien and Duffy, 2020; Strassman, 2020) and demonstrated benefits for both young scholars and experienced senior academics (e.g., Pérez-Mateo and Guitert, 2007; Bottoms et al., 2013; Tur and Urbina, 2016). For example, interdisciplinary research teams have more productive discussions on key issues and make decisions efficiently when members are in different geographical locations (Klonek et al., 2022). Interdisciplinary collaboration can be complicated, but essential for young scholars to develop their social networks and learn from senior academics (Rhoten and Andrew, 2004; Moore et al., 2018). In the collaborative process, diverse interdisciplinary team members could bring a broad range of skills and knowledge to help young scholars deal with complex issues (Gehlert et al., 2014; Graesser et al., 2018).

Compared to face-to-face interaction, digital technologies promote team collaboration through in-time networking, which is more affordable for university staff with limited budgets (Tur and Urbina, 2016). In addition, digital technologies enable both young scholars and senior academic staff to easily interact and share knowledge in virtual meetings; consequently, it might enhance team members' creativity and work productivity (e.g., Remmik et al., 2011; Stoszkowski et al., 2017). More recently, Lu (2020) examined WeChat as an innovative social media tool that could enhance European and Chinese academics' research collaboration in the humanities and social sciences (HSS). In this study, Lu proposed that the potential advantages and uses of social media tools could be explored in a broader way by interdisciplinary researchers.

Concerns about using digital technologies in virtual meetings have been also discussed in recent years. For example, during the pandemic, university faculty and students expressed concerns that looking at computer screens for prolonged periods of time could have a detrimental effect on mental and physical health (Bailenson, 2020; Biemans and Taghizadeh, 2023). Another study specifically looked at meeting fatigue via video conferencing (Fosslien and Duffy, 2020). Another concern is that the size of the computer screen might trigger “biochemical changes” and “physiological states” (Karl et al., 2022, p. 344) that are correlated with fight or flight (Dijker, 2014). Interdisciplinary senior academics often communicate via videos and voices but young team members might post their opinions via the chat box due to computer issues, content distraction, and low self-confidence (Wiederhold, 2020).

It is likely that interdisciplinary collaboration via virtual meetings will continue and might become the dominant communication method in the post-pandemic era. Because interdisciplinary collaboration is essential for knowledge production, this study aimed to examine both young scholars' and senior researchers' pain and joy when undertaking collaborative work while using digital technologies to communicate and meet. In the following sections, I will first review the relevant literature on knowledge production and then discuss media naturalness theory to provide a theoretical framework for understanding the inherent benefits and issues of using a virtual channel for communication.

## 2. Literature review

### 2.1. The new production of knowledge

In the 21st century, globalization has not only increased travel and immigration but also has brought about significant social change. Baber's *The New Production of Knowledge* maps “changes in the mode of knowledge production and the global impact of such transformations” (Baber, 1995, p. 751). Mode 2 (Gibbons et al., 1994) emphasizes a specific mode of scientific production in order to broaden knowledge sharing, transition, and collaboration—all of which are essential in society. This requires global academics to be more involved in the knowledge transition process and to also engage in a higher level of cooperation with their scientific peers. Mode 2 enables more people to be involved in the research process and improve their understanding of how science correlates with human movement. This new mode of knowledge production, which is reflexive, transdisciplinary, and heterogeneous (Gibbons et al., 1994), shows how these features connect with the changing role of knowledge in social relations. While the knowledge produced by research and development in science and technology is a central concern, Gibbons et al. (1994) outlined the changing dimensions of social science and humanities knowledge and the relation between the production of knowledge and its dissemination through education.

It is essential for young scholars and senior researchers to communicate and share information and knowledge in higher education. Lu (2020) believes that it is necessary for academics in scientific fields, such as Engineering, Architecture, and Mathematics, and those in Social Sciences to use social media technologies to enhance research collaboration. Studies show that social media tools are effective to improve HSS academics' research productivity and research collaboration across countries. More recently, a study (Haak et al., 2022) found that graduate students who were involved in a problem-based learning project were more motivated to engage with diverse stakeholders to drive transformational learning. Even though students were challenged to refine the conceptual model, they were able to develop a revised module conceptual framework that more accurately reflected the transdisciplinary nature of these interactions. However, most of these research studies were undertaken before the COVID-19 pandemic, and the interdisciplinary collaboration had occurred face-to-face. Little research has been conducted on completely virtual interdisciplinary collaboration.

### 2.2. Media naturalness theory

Based on Darwin's theory of evolution (Darwin, 1859), the human species survive and mate in a long process in which only the fittest and strongest offspring could live and further reproduce. This process thereby has enabled humans to propagate certain physical, behavioral, and cognitive traits (Kock, 2011). From an evolutionary view, co-located and synchronous communication has been the primary mode of communication for human beings, which means that humans are optimized for face-to-face interaction (Kock, 2009). Kock (2004) believes that face-to-face communication is a familiar mode for humans, which also means that a lower level of cognitive effort is required to use it. MNT identifies at least five key elements in human communication: (1) co-location, wherein individuals are present at a common place; (2) a high degree of synchronicity that allows individuals to exchange communicative stimuli quickly; (3) the ability to observe and express emotions through facial expressions; (4) the ability to observe and communicate through body language; and 5) the ability to use and listen to speech. Social media technologies can allow team members to communicate synchronously but it does not involve co-location and it is difficult to see facial expressions, especially if the video is small or unclear (Standaert et al., 2016, 2021). Consequently, virtual communication may feel unnatural and place more cognitive demands on users. Kock (2005) claims that individuals who used online media to perform collaborative tasks may achieve the same or better task-related outcomes than individuals using media with higher degrees of naturalness. However, others (Hantula et al., 2011) believe that MNT assumes that virtual communication could be too rich, leading to information overload, reduced productivity, and feeling overwhelmed. Recent studies (Torka, 2021) also found that virtual meetings were not that efficient for supervising teams online; that is, it is more difficult to sustain online team-based supervision than online one-on-one supervision as participants fail to adapt their interactions to the virtual format. Joylessness and meeting fatigue in staff are other points of criticism raised in recent scholarly work (Watson and Ireland, 2022).

### 2.3. Study context

This study is based on a teaching development program that aims to enhance interdisciplinary collaboration between academic staff and research students at X university, an international joint university in China. X university is an innovative joint university with a partnership in the UK. Compared with most Chinese public universities, X university has a large number of international staff, innovative teaching methods, and plenty of interdisciplinary collaboration. Moreover, staff at X university use a wide range of Western and Chinese digital technologies to collaborate in virtual meetings, such as Zhumu (Zoom), VooV, WeChat, university videoconference, and Microsoft Team. As virtual meetings are expected to become more common in the post-pandemic era, it is important to understand how senior researchers and young scholars engage in interdisciplinary collaboration via virtual meetings. As a variety of tools and technologies are used by staff and students, and they come from varied social and cultural backgrounds, it is assumed that their individual experiences, that is, the pains and joys they encountered in interdisciplinary collaboration during the pandemic might be different. More specifically, based on the literature review, we pursue the following research questions:

- What are the advantages offered by the use of digital technologies and virtual meetings in interdisciplinary collaboration?
- How do virtual meetings compare to face-to-face meetings?
- What are the major challenges faced by individuals in virtual meetings held for interdisciplinary collaboration (e.g., COVID-19, technology, and participant behavior)?

## 3. Research method

### 3.1. Research design and sample

Both young scholars (*N* = 14) and senior experienced researchers (*N* = 10) joined in the study. Among them, 14 are Europeans and 10 are Chinese. The data for this study were obtained from meeting minutes of 14 weeks of virtual meeting recordings in a semester (Zhumu, VooV, and Team), young scholars' reflections on their E-portfolios (University LM platform), and comments of meeting participants. All the texts were categorized into different topics, cut and pasted into an Excel Spreadsheet (Karl et al., 2022), such as “project application writing up,” “reading resource,” and “managing teaching schedule.” Texts (*N* = 503) obtained between May 2022 and January 2023 were included in the analysis.

To extract topics from the textual data, latent semantic analysis (LSA) in SAS Enterprise Miner was used. This is a powerful text-mining tool that uncovers underlying semantic concepts (i.e., topics) in a corpus. LSA is based on singular value decomposition, which is an extension of principal component analysis (Evangelopoulos et al., 2012). This is an appropriate method for understanding the thematic structure in textual data, and also for clustering and categorization. LSA has been widely used in different disciplines, such as computer-mediated communication (Cao et al., 2011; Xu, 2020), psychology (Arnulf et al., 2021), and quantitative reports and literature review (Jeyaraj and Zadeh, 2020). Hence, we decided that this method would be effective for uncovering the underlying topics related to participants' joy and pain in the virtual meeting environment.

First, the principal researcher preprocessed and cleaned the textual data by eliminating numbers and punctuations from the dataset. Moreover, based on Standard English stop word dictionary, the principal researcher excluded words such as “the,” “an,” and “a” from the dataset and reduced their dimensionality. Similar to Jeyaraj and Zadeh (2020), tokenization, lemmatization, stemming, spell-checking, and synonyms were examined in the process.

Second, following Shen and Ho (2020), a term-by-frequency matrix was created to parse the texts into a collection of terms. In a term-by-frequency matrix, each column of the matrix represents a unique word that appears across all textual data, and each row refers to each text. Each cell in the term-by-frequency matrix represents the number of times that a term (column) appears in a particular row (text). Using the term-by-frequency matrix, weighting alone cannot effectively distinguish different patterns of the textual data (Cao et al., 2011) because a term that appears commonly in a text may appear in other texts as well. For instance, in the virtual meeting recordings, the term “Zhumu” appeared in many texts, covering different topics or challenges related to Zhumu virtual meetings. To avoid such problems, the term frequencies were adjusted by the term frequency-inverse document frequency weighting scheme (TF-IDF).

To retain the intense and more meaningful topics in the data mining process, the principal researcher eliminated terms that appeared in less than four comments (Jeyaraj and Zadeh, 2020). The singular value decomposition (SVD) method was also adopted to reduce data dimensionality (Jeyaraj and Zadeh, 2020). The final step was to find the underlying dimensions linked to the theory in the LSA algorithm. As suggested by Evangelopoulos et al. (2012), the researchers used qualitative assessments to link the results to underlying theories. By using a qualitative content analysis method, researchers identified 12 possible topics in the corpus, using this number as the baseline to run the LSA algorithm. After the iterations in the qualitative analysis of categorizing texts, all the researchers agreed that the best degree of separation was when the LSA algorithm was run with five predefined topics.

## 4. Results

Five topic labels were identified after reviewing the topics in the rows. They are: *More effort and energy* (*n* = 132), *management skills* (*n* = 120), *technical knowledge* (*n* = 90), *unfamiliar field* (*n* = 84), and *discomfort* (*n* = 77). Participants' attitudes are shown in Table 1. The themes are presented in the following sections and tie the findings to the theory.

**Table 1:** Participants' attitudes toward the adoption of digital technologies in virtual meetings.

**Data source**	**Participants' attitudes**	**References**
Topics in virtual meetings, E-portfolio reflections, and comments via LM	•Positive/negative perceptions toward communication in the process of interdisciplinary collaboration during the pandemic	Positive (*N =* 245) •Increased communication opportunities •Synchronized communication •Saving research budget• Negative (*N =* 270) •Decreased communication efficiency •Lack of hands-on practice •Lack of rapport building •Lack of meeting management skills •Technical issues •Academic status concerns •Lack of family privacy
	•Positive/negative attitudes toward communication in the process of interdisciplinary collaboration before the pandemic period	Positive (*N =* 138)•Building mutual trust in a face-to-face format •Easy to communicate being in the same place •Only meeting during working hours• Negative (*N =* 97)•Increased budget for international travel •Try to avoid communication with people who are not of the same background
	•Recommendations for the post-pandemic era	•Meeting assistants required. •IT support/training •Individual time management

### 4.1. More effort and energy

The participants felt that they had to put more effort into virtual meetings than traditional face-to-face meetings. Sometimes, that would result in meeting fatigue and low energy. For instance, a young female scholar stated:

I am a young researcher (research student) and felt that I could not do much if many more experienced scholar were involved in the same meeting. Personally, I know that I am introvert and as a consequence, I am not that open to share information with senior researchers. I am always afraid to make mistakes and lose face. Sitting in a long virtual meeting, I could feel that a holder and some key persons always talk but the others keep silent most of time. It did not allow young scholars to be fully engaged. If this meeting were held face-to-face, I would sit close to some peers who might be research students as well. Then I could communicate with them more comfortably and confidently [SIC].

Interdisciplinary senior researchers also reflected that they were easily exhausted in the virtual meetings when sharing practical skills. A participant with a background in engineering commented:

When I presented a design model to the participants in virtual meetings, I found it difficult to interact as half of them were self-muted. I hardly knew people's reaction, not to mention their facial expressions. After finishing a virtual meeting, I need to grab a coffee immediately to refresh.

Others also expressed that they had concerns about joining meetings with someone that they have not collaborated with yet. Both senior researchers and young scholars prefer to have virtual meetings with people whom they already know or have some previous connections with at least. During the COVID-19 pandemic, they faced many challenges in their personal lives which made it harder to muster up the energy to communicate with people from their disciplines whom they did not know. Furthermore, a few young scholars thought that even though they put more effort into attending the virtual meetings, they obtained more information from other senior staff and peers. More specifically, they could send information via chatbox in the meetings, and gain constructive feedback synchronously.

### 4.2. Management skills

This theme included texts, comments, and reflections regarding meeting management skills and related issues, such as effective meeting schedules, participants' management, and multitasking in virtual meetings. Senior scholars believed that they needed to manage all the stakeholders in virtual meetings, including colleagues, young scholars, professional staff, and other invited guest speakers. They commented that they had to act as a “coordinator” rather than a single participant in most virtual meetings. For example, a senior scholar stated that it was difficult to keep everyone in the virtual meetings engaged, particularly, by using polls, document-sharing, and explaining questions in the chatbox. Young scholars also felt that they did not have any autonomy to check their availability before joining these virtual meetings as they mostly needed to follow the senior staff's schedule. This indicates that senior scholars had to manage many tasks including deciding the meeting dates, timelines, and number of participants, without any financial support.

Young scholars who engaged in interdisciplinary collaboration were eager to learn skills and obtain more experience from senior staff. However, due to the different time zones, some scholars had to sacrifice their spare time, such as late evenings after 10 p.m. or early mornings before 6 a.m., to join the virtual meetings. Moreover, they needed to undertake different tasks at the same time while joining the virtual meetings in the late evenings or early mornings. A young research student commented that she had a part-time job and it was hard to cope with so much multitasking in the virtual environment to finish collaborative work. A senior scholar shared his experience and made suggestions to enhance the effectiveness of virtual meetings in collaboration with others as follows:

I would like to share my research and teaching experience with young scholars, particularly, research students, and I strongly believe that it is essential for us to collaborate with junior staff. Then it might lead to more potential projects, but I am not good at managing group virtual meetings. As we know, these virtual meetings usually depend on individual research interests rather than group research funded work. Hence, there is no manager or administrative staff to organize meetings in advance. In that case, I have to learn how to set an agenda and goals for each meeting. Also, following up on those who might be interested in interdisciplinary collaboration via the virtual meetings is also time-demanding.

Some young research students noted that they would like to make contributions to setting up meeting agendas, supporting senior staff to collaborate with collaborators, and enhancing participant engagement. Meanwhile, they suggested that some young scholars could voluntarily work as meeting assistants to support the stakeholders in the virtual environment.

### 4.3. Technical knowledge

Some participants voiced that they were not good at using a range of technical tools during COVID-19, particularly, in a quarantine environment. A senior researcher mentioned that he felt frustrated when there was a 4-week lockdown in the town. He needed to reorganize all the meetings using different tools. For example, his collaborators preferred using Microsoft Team to hold virtual meetings, but Chinese partners could not use it. Therefore, he often needed to learn different meeting tools and then choose the most convenient one for stakeholders. However, technical information and knowledge do not seem to be a major challenge for young scholars in the collaboration process. A young male student commented as follows:

I am a born-digital generation and have gotten used to a range of digital products from an early stage. Personally, I think using a variety of digital technologies helped me become familiar with the virtual meeting environment, and also enhanced my digital skills and digital literacy. Take VOOV for example, Chinese researchers all used it via a Chinese Tencent, but it does not work for non-Chinese collaborators or someone who has not got a valid Chinese account. Before the meetings, I needed to give it a try by myself and report it to the meeting organizer if it did not work for my peers.

In addition, some participants believed that the different monitoring surfaces of various meeting software might be another challenge. For example, a small window for a chatbox could be in different places depending on the meeting software. Moreover, upload and download buttons could be in different shapes, which might be confusing for first-time users.

### 4.4. Unfamiliar field

Most young scholars reflected that they would struggle to be good icebreakers in virtual meetings with seniors or peers who were not in the same research field. Generally, scholars like sharing information and communicating with peers from their own fields because they have common interests and similar statuses (Wenger, 2010).

Young scholars found it difficult to interact with seniors in a virtual meeting if they were not from the same or a similar research field. They struggled to easily build rapport with senior staff to encourage smooth communication. Young researchers believe that a lack of rapport and comfort might easily make a virtual meeting an unpleasant or stressful experience. A junior staff with a background in humanity and social science stated:

I am interested in obtaining more knowledge from the interdisciplinary team members, but I have not received enough pre-meeting introduction of these seniors' backgrounds. After looking them up on the Internet by myself, I may feel that I am not familiar with the topics they work with and gradually become absent-minded in the meeting. This is very common for young research students, and many won't voice it. If the meeting is in a face-to-face format, I am able to see the person and they might be able to hold my attention even though the topic may be unfamiliar for me. For me, seeing a person in real life is different from meeting him/her in the screen (SIC).

### 4.5. Discomfort

Most of the comments on this theme focused on behavioral issues in the virtual meetings. For example, the virtual background setting in the meeting should be more professional rather than a cartoon one. Moreover, due to the virtual meeting setting limitations, some staff could not change their background to be a virtual picture so that the chaos in their room might be seen by other participants. A senior staff reflected that his discomfort was because some participants ate during the meeting. He felt that he found that disrespectful and unprofessional. He illustrated his negative experience:

The virtual meeting was around lunch time which was scheduled by an assistant. Half of the participants were having lunch in the meeting and the rest half did not turn on their cameras. Finally, I asked them to finish lunch first and then I would start to talk. It was so bad for me.

“I believe that if the meeting was booked at noon, it should be fine for participants to bring their lunch,” commented by a junior staff. A small number of participants did not like their children and family members to be seen in the background during virtual meetings in order to protect their family's privacy. Overall, the view was that it is reasonable for participants to bring their lunch if the meeting is scheduled at noon.

## 5. Discussion

To answer the first research question, the qualitative results show that most participants believed that virtual meetings (WeChat) were only effective for improving higher education management. That is, management expenses are definitely decreased as academics do not have to travel for conferences, demos, and practical procedures as all activities are held online. Thus, traveling costs are not included in the project budget. However, individuals might need more professional skills such as virtual meeting management skills and long-distance rapport building. These skills might be related to individual personality, cultural background, and quality of peer relationships (Lu et al., 2021) rather than interdisciplinary academic skills. Mejias (2007) showed that when influential members dominate a group discussion, it decreases young researchers' motivation in joining the meetings and lowers meeting satisfaction, and this trend has prevailed even in virtual meetings.

To address the second question, academics believe that in some ways virtual meetings were not better than face-to-face meetings. They felt that the perceived benefits are mostly in the area of institutional budget, meeting expenses, and personal costs. However, some young researchers believed that virtual meetings could be less effective as experimental practice benefits from face-to-face communication. These results align with MNT which emphasizes the importance of co-location and human physical expression (facial, visual, and movement) in the process of communication (Kock, 2004, 2009). In virtual meetings, people need to put more effort into preparation, completion of tasks, and interactions. Qualitative research does not support the findings of a quantitative study (Klonek et al., 2022) which found that virtual teams improved their team processes in the late pandemic period as compared to the early pandemic period. This might be due to individuals' motivation level, profession, and career stage when they were engaged in virtual meetings (Kasimoglu et al., 2022). The results also add a new insight to Mode 2, which has not been investigated with regard to knowledge production in a completely virtual environment.

To address the third question, challenges were influenced by three factors: technology, individual behavior, and research field. The text-mining results suggest that most participants' frustrations were focused on technical issues. Within these comments, over 78% were about the different versions of meeting software being installed (e.g., Chinese version, English version, and international version), widgets, tool functions, and switching between different versions. Another common comment was about the virtual meeting background and privacy (e.g., munching, kids, and family members). However, some participants critically commented that eating was not an issue as lunch meeting allows them to bring their food. Building a rapport is not easy, and young staff need to be more open to maintaining a communication channel to exchange ideas and share information with senior researchers. These results echo the finding that people need to exert more effort in speaking and listening in virtual meetings, particularly those who are less proficient in using social media tools (Standaert et al., 2016, 2021).

## 6. Practical implications

The pandemic has brought many uncertainties to academics, particularly, international and mobile researchers. The decreased research budget and limited opportunities to communicate in person have made interdisciplinary collaboration challenging. In an international university context, the adoption of various virtual meeting tools helped enhance disciplinary collaboration and research productivity during the pandemic. It is essential to provide academics, including seniors and young researchers, with technology training for using software and tools required for attending virtual meetings, thereby minimizing their job-related stress during the pandemic (Rogelberg et al., 2006; Cheng et al., 2016; Lehmann-Willenbrock et al., 2018). These trainings and workshops should not only introduce the key features of the technology tools and platforms for virtual meetings (e.g., Zoom, Microsoft Teams, and Tencent) but also show demos of new features of these tools or apps that help enhance meeting effectiveness. For example, the international version of Tencent (VooV) needs more explanations for international researchers who have never used a Chinese version.

Moreover, the results suggest that academics may wish to consider how to set boundaries to maintain work–life balance. International academics working in local universities should be informed about expectations regarding how researchers should behave in virtual work meetings. In addition, senior researchers should ask for help from the faculty if they do not have personal assistants to help them set up virtual meeting facilities. In addition, they must consider attending virtual workshops to obtain guidance regarding using the necessary software. School managers and leadership roles are responsible for reinforcing the importance of these workshops/training sessions, as well as structuring the virtual meeting behavior. In this study, the findings also suggest that virtual meetings can lead to job fatigue and negative consequences. Therefore, organizations must carefully decide upon the frequency and length of virtual meetings.

## 7. Limitations and future research

Although this study yielded interesting results, it has three notable limitations. First, this study only focused on academic staff who have worked in an international university in China. The sample and the working climate might be different for public universities. Second, the data were mainly textual data, including comments, meeting record transcriptions, and personal reflections. In future, we will consider using a mixed research design to enhance the data resources. A mixed research design might help us understand how to enhance the work satisfaction and engagement of academics and the effectiveness of virtual meetings to ultimately improve research communication. Third, a snowball recruitment technique might have excluded participants who are shy and introverted but have a strong motivation for joining such research studies. In future, we might use a random sampling technique to recruit more suitable participants.

In this study, we found that work–life balance is essential for researchers in the pandemic. Future research could focus on examining if there is any gender difference in virtual meetings. Women might find it more difficult to balance work and life in the pandemic and post-pandemic era. Moreover, researchers could use social network analysis to explore more deeply the ways in which interdisciplinary teams collaborate in virtual environments. This might lead us toward further investigation from a cross-cultural perspective.

## Data availability statement

The original contributions presented in the study are included in the article/supplementary material, further inquiries can be directed to the corresponding author.

## Ethics statement

Ethical approval was provided for this study on human participants by Xi'an Jiaotong-Liverpool University. The patients/participants provided their written informed consent to participate in this study.

## Author contributions

JL conceptualized the work, finished the data collection and analysis, and completed the article writing.
